# Factores que interfieren en el resultado de la HbA1c

**DOI:** 10.1016/j.aprim.2025.103292

**Published:** 2025-06-26

**Authors:** Marta Ballesteros Merino, Rosalina Martínez López, Laura Navarro Casado

**Affiliations:** Servicio de Análisis Clínicos, Complejo Hospitalario Universitario de Albacete, Albacete, España

La hemoglobina glicada (HbA1c) es un criterio diagnóstico de diabetes y prediabetes, asimismo el principal biomarcador del control glucémico y pronóstico en el diabético tipo 2. La hemoglobina presente en los hematíes está conformada por el grupo hemo y 4 cadenas de globina (α, β, δ o γ), que determinan el tipo de hemoglobina. El 97% de la hemoglobina del adulto corresponde a hemoglobina A (ααββ). Mediante una reacción lenta y no enzimática, la glucosa puede unirse a puntos diferentes de la hemoglobina A. El 80% de la glicación tiene lugar a nivel de la valina del extremo N-terminal de la cadena β, que es lo que se conoce como HbA1c, fracción medida e informada desde el laboratorio. Su formación va a depender de la concentración de glucosa a la que se exponga el hematíe durante su vida útil (90-120 días), correlacionándose mejor con la glucemia media de los 2-3 últimos meses^1^. La precisión de la medición está condicionada por el método utilizado. El laboratorio clínico conoce sus limitaciones y es el responsable de informar de las interferencias, principalmente hemoglobinopatías, que dificultan o impiden el análisis^2^, ^3^. Variaciones mínimas pueden explicarse por la variabilidad biológica intraindividual^4^. Sin embargo, diversos factores pueden interferir de forma clínicamente relevante en la medida de la HbA1c.

## Caso 1

Mujer de 54 años diabética con HbA1c del 6,4% en control previo semestral. Se solicita analítica por astenia, que evidencia: hemoglobina (Hb) 8 g/dl; LDH 499 U/L; bilirrubina total 1,9 mg/dl; haptoglobina < 3 mg/dl; glucosa 176 mg/dl y HbA1c del 2,6%. Ingresa con diagnóstico de anemia hemolítica. Resuelta la hemólisis al mes, se objetiva: Hb 14,2 g/dl; glucosa 135 mg/dl y HbA1c del 4,7% y a los 2 meses: glucosa 128 mg/dl y HbA1c del 7%, siendo acorde con su control habitual.

## Caso 2

Varón de 76 años diabético, con glucosa de 176 mg/dl y HbA1c del 5,6% tras haber recibido transfusión sanguínea por anemización aguda secundaria a úlceras gástricas hace 10 días. En el control cardiovascular del mes previo presentaba: glucosa 196 mg/dl; Hb 14,1 g/dl y HbA1c del 9,8%. Hasta pasados 3 meses la transfusión impide la correcta interpretación de la HbA1c.

**Figura 1:**
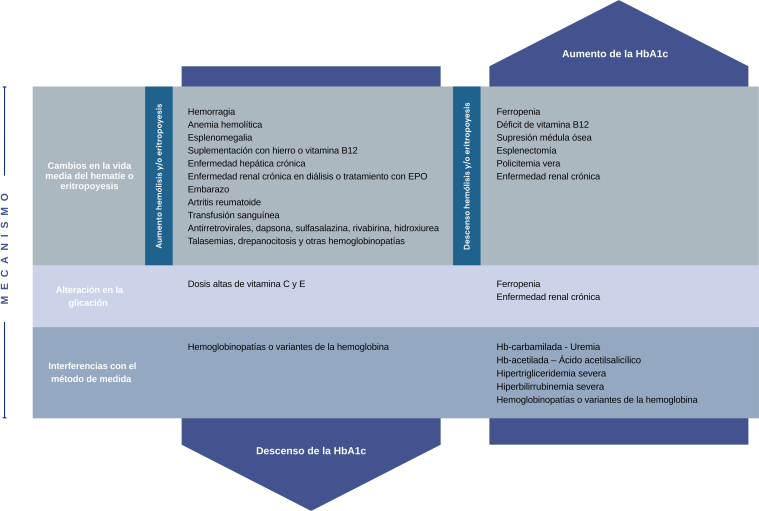
Factores y mecanismos que interfieren en el resultado de la medición de la HbA1c.

Todas las condiciones que determinan cambios en la vida media del hematíe (alteración de eritropoyesis o hemólisis), provocarán un mayor o menor tiempo de exposición a la glucosa, resultando HbA1c falsamente elevada o disminuida respectivamente. Otros factores indicados en la figura 1, también pueden alterar la fiabilidad de la medida^1^, ^2^, ^3^, ^5^, ^6^. Los casos descritos reflejan valores de HbA1c erróneamente bajos. En el primero de ellos provocado por la lisis aumentada de los hematíes y en el segundo por la transfusión, que produce un aporte de hematíes no glicados y un efecto dilucional^5^, ^6^.

La evaluación de la HbA1c previa, la no correlación entre la HbA1c determinada con el contexto clínico y/o la glucemia, ayudan a pensar en la posibilidad de que exista algún factor interfiriendo en su resultado.

## Conclusión

Los casos presentados demuestran que no siempre la medida de la HbA1c refleja adecuadamente el control glucémico, lo que puede conllevar la toma de decisiones inapropiadas. El clínico debe conocer los potenciales factores que interfieren en los valores de la HbA1c. Por tanto, no debería interpretarse de forma aislada, sin antes tener en cuenta las condiciones del paciente y considerar métodos alternativos para su manejo adecuado.

## Financiación

El estudio no ha recibido financiación externa.

## Consideraciones éticas

En el estudio se han seguido los protocolos institucionales sobre la publicación de datos de pacientes, se ha respetado su privacidad.
